# The minimal perceived change: a formal model of the responder definition according to the patient’s meaning of change for patient-reported outcome data analysis and interpretation

**DOI:** 10.1186/s12874-021-01307-9

**Published:** 2021-06-21

**Authors:** Antoine Vanier, Véronique Sébille, Myriam Blanchin, Jean-Benoit Hardouin

**Affiliations:** 1 Inserm-University of Tours-University of Nantes, UMR U1246 Sphere “Methods in Patient-Centered Outcomes and Health Research”, 37000 Tours, France; 2 Inserm-University Hospital of Tours, CIC 1415, Unit of Methodology-Biostatistics Data Management, 2, Boulevard Tonnellé, 37000 Tours, France; 3 University Hospital of Nantes, Unit of Methodology and Biostatistics, 44000 Nantes, France

**Keywords:** Clinical research, Patient-Reported Outcomes, Minimal Clinically Important Difference, Minimal Important Difference, Responder Definition, Estimation, Theory, Model, Psychometrics, Health-Related Quality of Life

## Abstract

**Background:**

Patient-Reported Outcomes (PROs) are standardized questionnaires used to measure subjective outcomes such as quality of life in healthcare. They are considered paramount to assess the results of therapeutic interventions. However, because their calibration is relative to internal standards in people’s mind, changes in PRO scores are difficult to interpret.

Knowing the smallest value in the score that the patient perceives as change can help. An estimator linking the answers to a Patient Global Rating of Change (PGRC: a question measuring the overall feeling of change) with change in PRO scores is frequently used to obtain this value. In the last 30 years, a plethora of methods have been used to obtain these estimates, but there is no consensus on the appropriate method and no formal definition of this value.

**Methods:**

We propose a model to explain changes in PRO scores and PGRC answers.

**Results:**

A PGRC measures a construct called the Perceived Change (PC), whose determinants are elicited. Answering a PGRC requires discretizing a continuous PC into a category using threshold values that are random variables. Therefore, the populational value of the Minimal Perceived Change (MPC) is the location parameter value of the threshold on the PC continuum defining the switch from the absence of change to change.

**Conclusions:**

We show how this model can help to hypothesize what are the appropriate methods to estimate the MPC and its potential to be a rigorous theoretical basis for future work on the interpretation of change in PRO scores.

## Background

In the context of human healthcare, *Patient-Reported Outcomes* (PROs) are standardized instruments, mostly in the form of self-administered questionnaires, that are increasingly used to measure relevant concepts (or *constructs*, in psychometric language) that are best assessed through patients’ speech or thoughts [5, 19]. Constructs measured by PROs can include pain, fatigue, anxiety, depression, health status or Health-Related Quality of Life (HRQoL). PROs are on either a unidimensional *scale* (unidomain construct) or a *profile* of multiple scales (multidomain constructs), each measured by one or multiple *items* (i.e., questions) with a prespecified response format (e.g., a *Likert scale* or a *Visual Analogous Scale* (VAS)) [5]. For a given scale, the measure of the construct (e.g., level of fatigue), a single quantitative value, is obtained by an algebraic transformation of the responses to item(s), called the *measurement model* [37]. The measurement model can be as simple as summing the responses to items or can be more complex. These measures are used in clinical research to assess the effectiveness of newly developed therapeutic interventions, in epidemiology to survey health status at a populational level, and in healthcare to monitor the evolution of patients’ state over time [5]. In recent years, the systematic use of PROs in healthcare has been advocated because they are instruments designed to assess patients’ experience, feelings or preferences regarding their treatment course [23, 43]. Thus, in a paradigm of shared decision making in healthcare, PRO measurements are frequently viewed as paramount endpoints (e.g., HRQoL) to consider patients as beings with unique subjective experiences rather than merely as objects with a presence in physical reality [43].

As instruments designed to quantitatively measure constructs, a PRO must comply with a sufficient level of measurement properties to be useful. It must be *valid* (i.e., it must measure what it intends to measure) and *reliable* (i.e., it must be free of *measurement error*) [14]. Currently, a high number of PROs are regarded as having achieved these prerequisites [5]. Nonetheless, users of PROs (e.g., health care professionals, clinical researchers, policy-makers, patients) still face issues when interpreting a change in scores over time (i.e., extracting relevant meaning that allows practical decision-making) [37]. Two broad explanations can be hypothesized to explain this difficulty. First, PRO scales have a much shorter history of development than the International System (IS) in physics and are less widespread than other types of measurement (e.g., biological values) in routine healthcare practice. Therefore, the process of attaching meaning to changes in PRO scores over time is still developing in the healthcare community. Second, when calibrating a PRO scale, which is usually expressed on an arbitrary continuum (e.g., an interval from 0 to 100; sometimes the interval is the real line), the *unit of measurement* is frequently defined relative to a subjective phenomenon. For instance, a scale measuring pain intensity on a 100-unit VAS is dependent on a subjective interpretation of an “absence of pain” and “the worst pain you can imagine” to define its bounds. Additionally, the change in pain intensity that is required to define one unit of measurement is relative to an internal standard in people’s minds [26]. Thus, the meaning of an improvement of, for instance, 4 points on a 100-unit scale of HRQoL after a therapeutic intervention can be difficult to comprehend (at both the group and the individual level). Aside from the issue of reliability (disentangling *true change* from measurement error), the question of the practical relevance of a level of change for the patient frequently arises [30].

To enhance the interpretability of PROs, the US Food and Drug Administration (FDA) proposed in 2009, in the context of clinical research, the use of an a priori* Responder Definition* (RD) which is defined as “*the individual Patient-Reported Outcomes (PRO) score change over a predetermined time period that should be interpreted as a treatment benefit*” [32]. The RD can be used as a threshold value to classify each patient as having experienced or not experienced a minimal treatment benefit. For a given PRO scale, the RD value can be obtained through a choice of numerous perspectives [1]. It can be a value linking PRO change relative to the patient’s perspective by using medical outcomes with clinical relevance, such as disease severity, level of symptoms, response to treatment, prognosis or functional impact (e.g., the minimum change in PRO scores associated with a specific gain in motor function [3, 41]. It can also be a value linking PRO change relative to the healthcare professional’s perspective (e.g., the minimum change in PRO scores a medical doctor classifies as beneficial) or to the societal perspective (e.g., health care utilization, decrease in mortality or morbidity) [1, 3]. In addition to these perspectives, one that has been extensively studied in the last 30 years is to obtain an RD value by linking PRO change to the subjective meaning of a relevant change for a patient. This approach is frequently called the *Minimal Clinically Important Difference* (MCID) or *Minimal Important Difference* (MID) and was proposed by Jaeschke et al. in 1989 [9]. Its initial definition is as follows: “*the smallest difference in score in the domain of interest which patients perceive as beneficial and which would mandate, in the absence of troublesome side effects and excessive cost, a change in the patient's management*” [9]. Estimating the MID of a given PRO has been the subject of hundreds of studies [36].

Several methods have been proposed for MID determination and are mainly classified as *anchor-based* and *distribution-based* methods [24]. Anchor-based methods imply a principle that involves the use of an external indicator (*the anchor*) to classify patients as improved or worsened and to link this to the change in scores. For instance, the most commonly used anchor is a *Patient Global Rating of Change* (PGRC) [36]. This is a single item used at a second time of measurement (e.g., at the end of a therapeutic intervention) that asks the patient to make an overall judgment of his/her level of change in the construct of interest since a relevant reference point (e.g., the beginning of the therapeutic intervention) [9]. For instance, the PGRC can be phrased as follows: “*Since the beginning of your treatment, overall, do you think your quality of life is now…*”. Proposed responses can be “*a lot worse*”, “*a little worse*”, “*about the same*”, “*a little better*”, and “*a lot better*”. By using the distribution of responses of people who answer that they have changed slightly and linking it to the observed change in PRO scores, an MID value can be determined. Numerous estimators have been proposed, but a simple and frequently used one is to take the sample mean in the PRO change in scores of people who classify themselves as slightly changed [24, 36]. Although numerous issues have been raised about anchor-based methods using a PGRC (e.g., the validity and reliability of the PGRC, issues of recall bias) [12], these methods are frequently viewed as appropriate to estimate an MID because they are estimates with a design linking the change in PRO scores to the subjective meaning of change according to patients [30]. In contrast, distribution-based methods use the variability of the overall PRO score(s) to estimate the MID without any explicit assessment of the perception of change by the patient [24]. Two approaches are most common. The first is to use Cohen’s rules, known as *Effect Sizes* (ES). An ES is obtained by dividing the mean change in scores by the standard deviation of the baseline score [4]. Based on observations in psychological and social sciences, Cohen empirically defined an ES of 0.2 as small, 0.5 as moderate (a change that can be detected by the human “naked eye”) and 0.8 as large [4]. Thus, a change in PRO scores of 0.5 ES is frequently used as an estimation of the MID [17, 36]. Another approach is to relate changes in PRO scores to measurement error [24]. For example, based on empirical observations, some authors argue that 1 *Standard Error of Measurement* (SEM) can approximate an appropriate MID value [40, 42].

Over the last 30 years, a plethora of methods have been proposed and used to estimate the MID of a PRO [36]. Investigating the RD threshold from different perspectives (e.g., the societal threshold and the individual threshold) can help to obtain complementary and useful information to enhance the interpretation of a change in PRO scores over time. Nonetheless, from the patient perspective, especially with regard to the perception of change by the patient (i.e., obtaining an MID value), there is currently no consensus on the adequate method(s), and there is little to no knowledge about the statistical properties of the proposed estimators (e.g., *bias* against a *true populational* MID value). Thus, the current guideline is the use of “*triangulation*”, which is the use of multiple estimators from different types of designs (e.g., anchor-based designs and distribution-based designs) to obtain the plausible range of the MID value for a given PRO [10]. However, it seems unreasonable to think that all the estimators proposed for determining an MID according to the patient’s subjective perspective of change are appropriate. In a recent study to determine the MID of the *General Health* domain of the *SF-36* (a common PRO used to assess HRQoL), the resulting MID value ranged from 1 to 26 on a 100-unit scale according to the different proposed estimators [39].

Despite the need to identify useful methods to extract relevant data to enhance PRO interpretability, this field of research is hampered by a lack of formal clarity. Specifically, one of the paramount issues is the absence of a formal definition of the MID value as a *statistical parameter* with a known definition in the *population*. In *estimation theory*, it is essential to have a rigorous definition of a parameter in the population (e.g., the expected value) to assess the properties of a proposed *estimator* (e.g., the sample mean). However, for the past 30 years, MIDs have been estimated on empirical data without a definition of this parameter in the population [36].

To make progress on the issue of defining and assessing the statistical properties of relevant methods to estimate the MID of a PRO, the main objective of this paper is to propose a formal model and definition of the MID as a statistical parameter in the population. A second objective of the paper is to show that with this proposed definition, we will be able to deduce hypotheses about relevant method(s) to estimate an MID.

First, within the general issue of interpretability, we set the frame of our work. Second, we introduce the Rapkin and Schwartz model [22], a model initially designed to describe the components that explain change in HRQoL over time, which will be the basis of our model. Third, we propose a modified and expanded version of Rapkin and Schwartz’s model to illustrate the components engaged when someone must rate his/her level on a given PRO at two times of measurement and answer a Patient Global Rating of Change at the second measurement. Fourth, from this model, we propose a formal definition of an MID according to the patient’s meaning of change as a statistical parameter in the population. Fifth, we propose hypotheses about an adequate method(s) to estimate this value in a sample. Finally, we discuss the limits and perspectives of this proposal.

## Methods

### Frame: The minimal perceived change

As mentioned above, multiple perspectives can be considered when estimating an RD value for a given PRO [1]. For example, a modest mean improvement in an outcome for a common condition may be relevant at a societal level, while this same level of improvement may be meaningless at an individual level [25]. Patients and healthcare professionals frequently have different expectations about the outcome of a therapeutic intervention [38]. In addition, the concept of the MID has generated various debates about its definition and relevance. Some of these issues involve the distinction between obtaining information on a threshold that characterizes a change as “minimal” *versus* “meaningful” or the non-ambiguous meaning of “important” [24, 30]. The FDA Patient-Focused Drug Development Guidance Public Workshop of 2018 states that “the minimum change may not be sufficient to serve as a basis regulatory decisions” [33]. Therefore, the FDA advocates the use of anchor items that include meaningful and useful response options. Nonetheless, what is considered “meaningful” from the patient’s perspective can be difficult to capture without ambiguity given the polysemy of the term. Other issues involve the need for the MID concept to embody a sufficient level of change, such as mandating a change in therapeutic management or incorporating a perspective with clinical implications (hence the debate about MCID *versus* MID) [30]. These considerations indicate that defining an RD threshold value that encompasses all perspectives is untenable. Thus, we first need to explicitly frame our proposed definition of an RD value.

The perspective we choose is the patient’s perspective. More specifically, our RD threshold value concerns the patient’s subjective meaning of what he/she considers change. Thus, for the remainder of the manuscript, the theoretical model we develop to define an RD threshold value corresponds to the minimal amount of change in PRO scores that is subjectively considered a change by a patient. We call this the “*Minimal Perceived Change*” (MPC) and its definition is “*the minimum amount of change in PRO scores over time that is perceived by a person as a nonstable trajectory*”. We acknowledge that by focusing on the minimum perceived change, our approach could be considered contradictory to the recommendation of the FDA to provide results on “meaningful” change. However, we argue, as other authors have [30], that providing results on minimal perceived change from the patient’s perspective has meaning. Moreover, we believe that defining minimal perceived change is less ambiguous than “meaningful change”. We emphasize that we do not claim that our theoretical proposition encompasses all perspectives regarding the issue of PRO interpretability.

### Introducing the Rapkin and Schwartz model of appraisal

To develop a formal definition of the MPC as a statistical parameter in the population, there is a need for a conceptual model that describes the components that are engaged when someone answers a PRO and a PGRC item at two times of measurement. The model we propose is an adapted and expanded version of the Rapkin and Schwartz model of appraisal [22]. Thus, we briefly present this appraisal model.

**Fig. 1:**
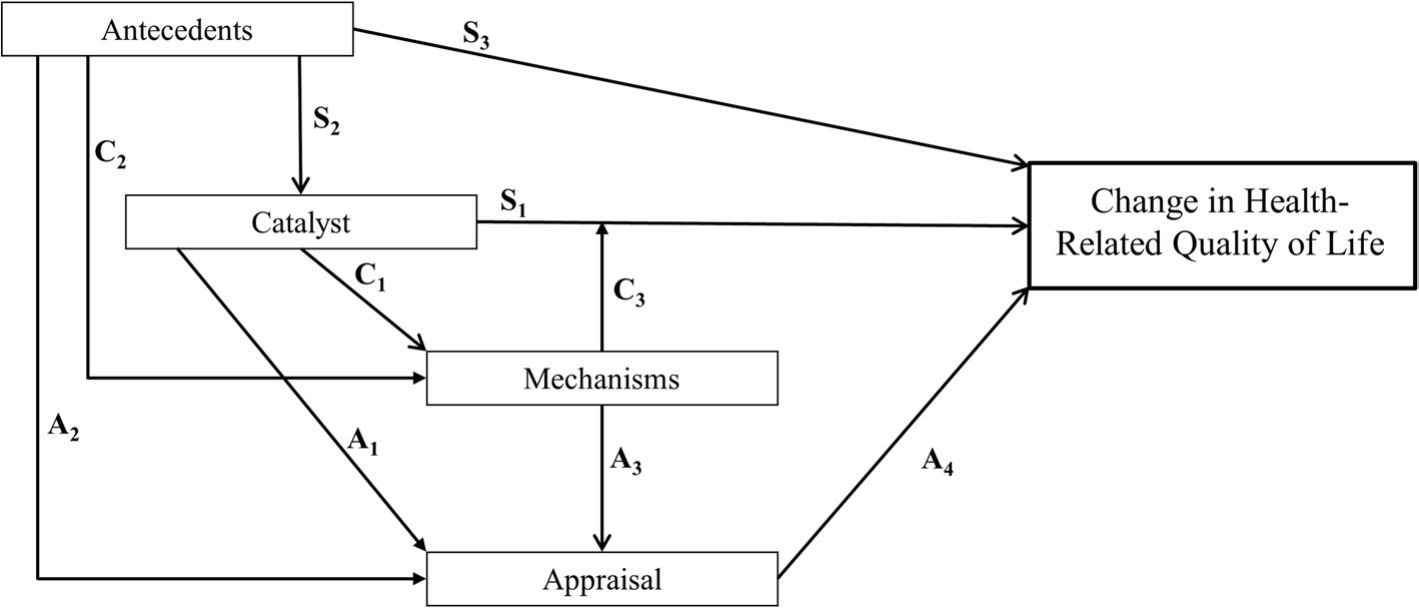
An adapted version of the original Rapkin and Schwartz model explaining change in HRQoL. Accounting for changes in standard influences (S), coping processes (C), and appraisal processes (A). Adapted from Rapkin and Schwartz, *Health and Quality of Life Outcomes*, 2004 [22]

The Rapkin and Schwartz model was published in 2004 (an adapted version for this paper is depicted in Fig. 1). The model was initially designed to explain change in HRQoL, but it can be understood as a model describing the components that explain change in a construct of interest to be measured with a PRO in the context of health-related research or healthcare.

Briefly, this model focuses on a *catalyst* (i.e., a salient health event such as the diagnosis of a disease) to directly explain part of the change in HRQoL. The occurrence of the catalyst is set by *antecedents* (i.e., stable or dispositional characteristics of the individual, such as socioeconomic status), which also directly explain part of the change in HRQoL. Moreover, the occurrence of the catalyst triggers psychological *mechanisms* (i.e., behavioral, cognitive and affective processes such as coping strategies), which allow psychological adaptation to illness by directly buffering the catalyst or through an indirect path mediated by changing the way the construct of interest is appraised (i.e., the *appraisal* processes: the cognitive processes engaged when someone must answer a PRO item) [31]. The model is fully detailed in Supplementary Appendix I.

## Results

### A first proposal: a model explaining the perceived change

The conceptual model we propose is an adaptation and expansion of the Rapkin and Schwartz model. Its goal is to model the components and relationships engaged when an individual answers a PRO at two times of measurement and answers a PGRC at the second time. This model is a *Structural Equation Metamodel* [7]. It is depicted as a Directed Acyclic Graph [6]. The variables engaged are depicted as broad concepts regardless of whether they can be assessed by a single or multiple item(s) and regardless of whether they are *manifest variables* (i.e., directly observed in empirical data) or *hypothetical latent variables*. Finally, the model depicts only structurally directed relationships regardless of the mathematical functions that can be used to model links (including the choice of linear or nonlinear functions). Because it models a longitudinal process, the passage of time drives the relationships (effects cannot precede cause). As such, the model is intended to include *unidirectional relationships* (cause-effect relationships) as much as possible. Nonetheless, *bidirectional relationships* (correlations) are considered between simultaneous processes for which a directed sense of causality cannot be determined based on theoretical and experimental arguments. For simplicity, the model depicts only direct or mediated relationships between the components and does not represent interactions. However, interactions can exist.

The first step that was required to adapt the Rapkin and Schwartz model to the current issue was to develop the model to represent two measurements and to generalize the notion of HRQoL to any construct of interest measured by a PRO. The original model was initially developed to explain changes in HRQoL; as such, the outcome is the change. Thus, it is not a longitudinal model that explicitly represents two measurements.

**Fig. 2:**
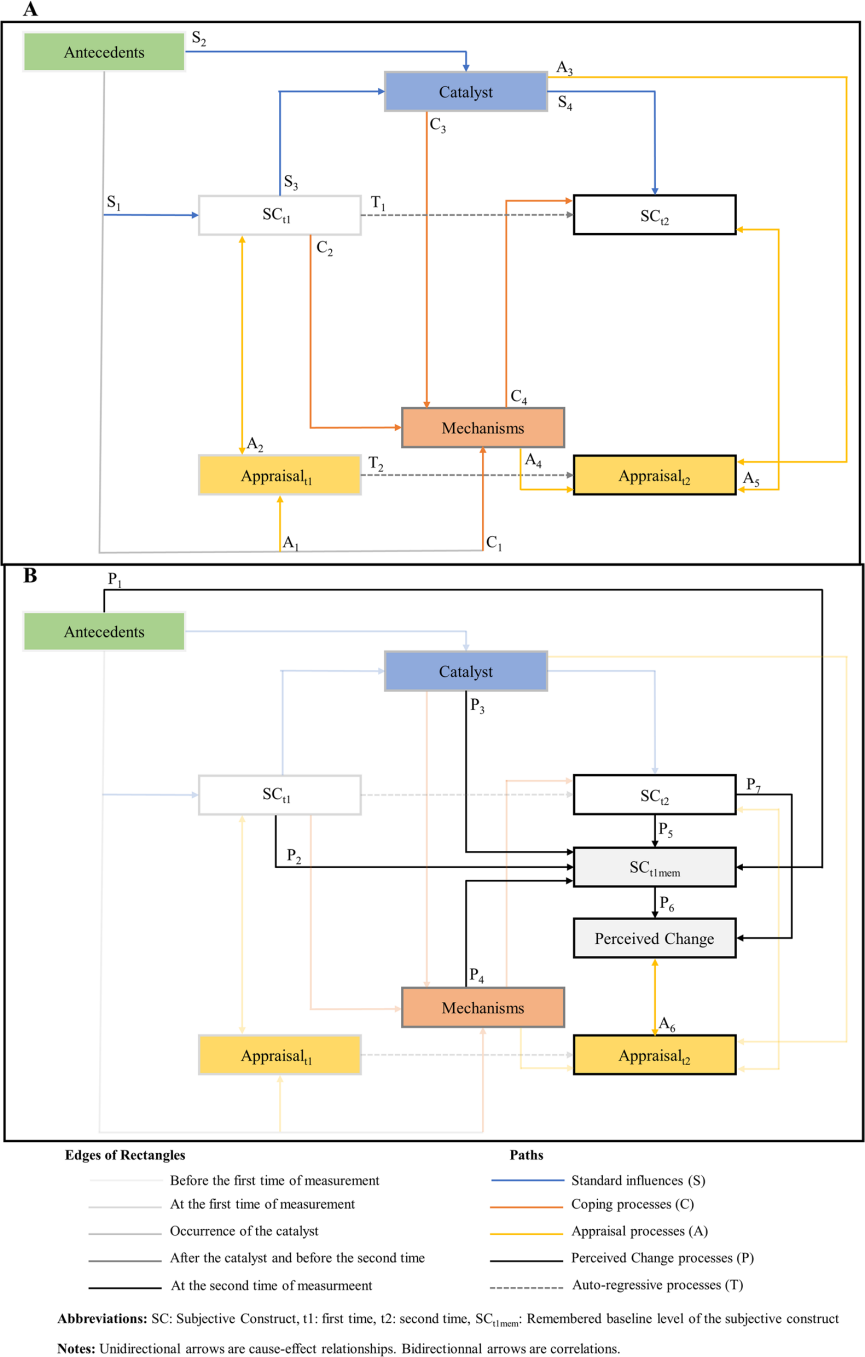
Building a theoretical model explaining perceived change. **a**: Expanding and generalizing the Rapkin and Schwartz model for two measurements and any subjective construct of interest. **b**: Adding necessary concepts and paths to explain perceived change

The result of this first step is depicted in Fig. 2a. This intermediate model is a representation of the Rapkin and Schwartz model that is developed to represent two measurements. Time passes from left to right, and the different components are placed to represent their relative occurrence in time (the borders of the rectangular boxes go from light gray (e.g., *Antecedents*) to dark (e.g., *Appraisal*_*t2*_) to reflect the passage of time). Antecedents, catalysts, and mechanisms are present, as in the Rapkin and Schwartz model. However, instead of a change in HRQoL, there are now *SC*_*t1*_ and *SC*_*t2*_, two concepts representing the subjective construct of interest that is measured at two times, along with *Appraisal*_*t1*_ and *Appraisal*_*t2*_*,* the appraisal processes engaged when rating SC_t1_ and SC_t2,_ respectively. Blue arrows (“*S*” paths) are the relationships corresponding to the “standard” influences of the Rapkin and Schwartz model (e.g., the direct effect of the catalyst on the construct of interest at time 2). We can note the addition of arrow *S*_*3*_ (Fig. 2a) to incorporate the possibility of an influence of the level of the target construct at time 1 on the catalyst (e.g., at time 1, the subject has a high level of fatigue, which can have an effect on the probability of a car accident, the catalyst). We can also note the addition of the *C*_*2*_ arrow (Fig. 2a) to account for an influence of the level of the target construct at time 1 on the mechanisms (e.g., at time 1, the subject has a high level of fatigue, which can have an effect on the type of psychological mechanisms the subject can elicit to accommodate the effect of the catalyst). Orange arrows (“*C*” paths) represent relationships pertaining to the occurrence of *coping strategies* triggered by the occurrence of the catalyst. For simplicity, we choose to represent the influence of the *psychological mechanisms* on the level of the target construct as an effect pointing directly to SC_t2_ (*C*_*4*_ path) rather than an effect buffering the path between the catalyst and SC_t2_ (as in the original Rapkin and Schwartz model). We believe that if such an effect is more relevant for inclusion as a buffer effect, it would be a matter of appropriate mathematical modeling, which is not the focus here. Yellow arrows (“*A*” paths) represent relationships related to the *appraisal processes* engaged at the two times of measurement. At each time of measurement, the rating of the corresponding target construct invokes a set of appraisal processes in an individual’s mind (*A*_*2*_ and *A*_*5*_ paths, respectively). However, it can also be hypothesized that the level of the target construct at each measurement time has an influence on the set of appraisal processes engaged; hence, the bidirectional arrows (e.g., the appraisal processes engaged to answer PRO item(s) with a high level of fatigue will not be the same as if the subject feels rested). The two dashed arrows (“*T”* paths, T for time) represent the possibility that even after accounting for the other paths in the model, some of the variance of SC_t2_ can only be captured by the variance of SC_t1_ (autoregressive path *T*_*1*_), and some of the variance of *Appraisal*_*t2*_ can only be captured by the variance of *Appraisal*_*t1*_ (autoregressive path *T*_*2*_).

The second step that was required to expand the original appraisal model was to add two new cognitive components to account for the processes engaged when answering a PGRC at a second measurement time. We hypothesize that when patients must answer a PGRC, they must form in their mind an idea of its perceived change by comparing their level of the subjective construct at the first time and its current level (i.e., at the second time of measurement). Thus, we hypothesize that it is necessary to add two supplementary cognitive components to the model: the *Perceived Change* (*PC*) and the *remembered baseline level of the subjective construct* (*SC*_*t1mem*_).

The result of this second step is depicted in Fig. 2b. The new paths resulting from these additions are the “*P*” paths (perceived change paths). Perceived change is the overall feeling of change from the patient’s first experience to the second measurement. It is the difference between (and therefore a function of) the remembered baseline level of the subjective construct (i.e., SC_t1mem_, *P*_*6*_ path) and the level of the subjective construct at the second time (i.e., SC_t2_, *P*_*7*_ path). The remembered baseline level of the subjective construct (SC_t1mem_) is a level the subject forms or extracts from memory that represents the level of SC_t1_ in comparison with the level of SC_t2_ to produce the PC. SC_t1mem_ is a function of SC_t1_ (*P*_*2*_ path) and SC_t2_ (*P*_*5*_ path) because we wanted to represent two potential cognitive processes that may be at play when someone is asked to form a retrospective assessment of the level of a target construct. The P_2_ path represents the agreement between SC_t1mem_ and SC_t1_, or the capacity of a patient to extract from memory an accurate value of his/her level of the subjective construct at baseline. The P_5_ path represents the agreement between SC_t1mem_ and SC_t2_, or the tendency of a patient to reconstruct his/her level of subjective construct at baseline as an inference starting from his/her present state (i.e., SC_t2_). This latter cognitive process is also known in the literature as the “*implicit theory of change*” [16]. To date, the magnitude of both processes when formulating a retrospective judgment is unknown, but there is empirical evidence that both can be at play [13, 15]. Other contingencies can influence the level of SC_t1mem_, such as the antecedents (*P*_*1*_ path, e.g., the overall cognitive ability of the patient), the catalyst (*P*_*3*_ path, e.g., a traumatic event resulting in memory impairment), and the psychological mechanisms (*P*_*4*_ path, e.g., a coping strategy when the patient underestimates his/her baseline level of HRQoL (and therefore his/her decrease in HRQoL over time) to better adjust with the anxiety of declining). Finally, the patient is asked to rate his/her PC by answering a PGRC at the second measurement; hence, the bidirectional *A*_*6*_ path between the appraisal processes at the second measurement and the PC.

**Fig. 3:**
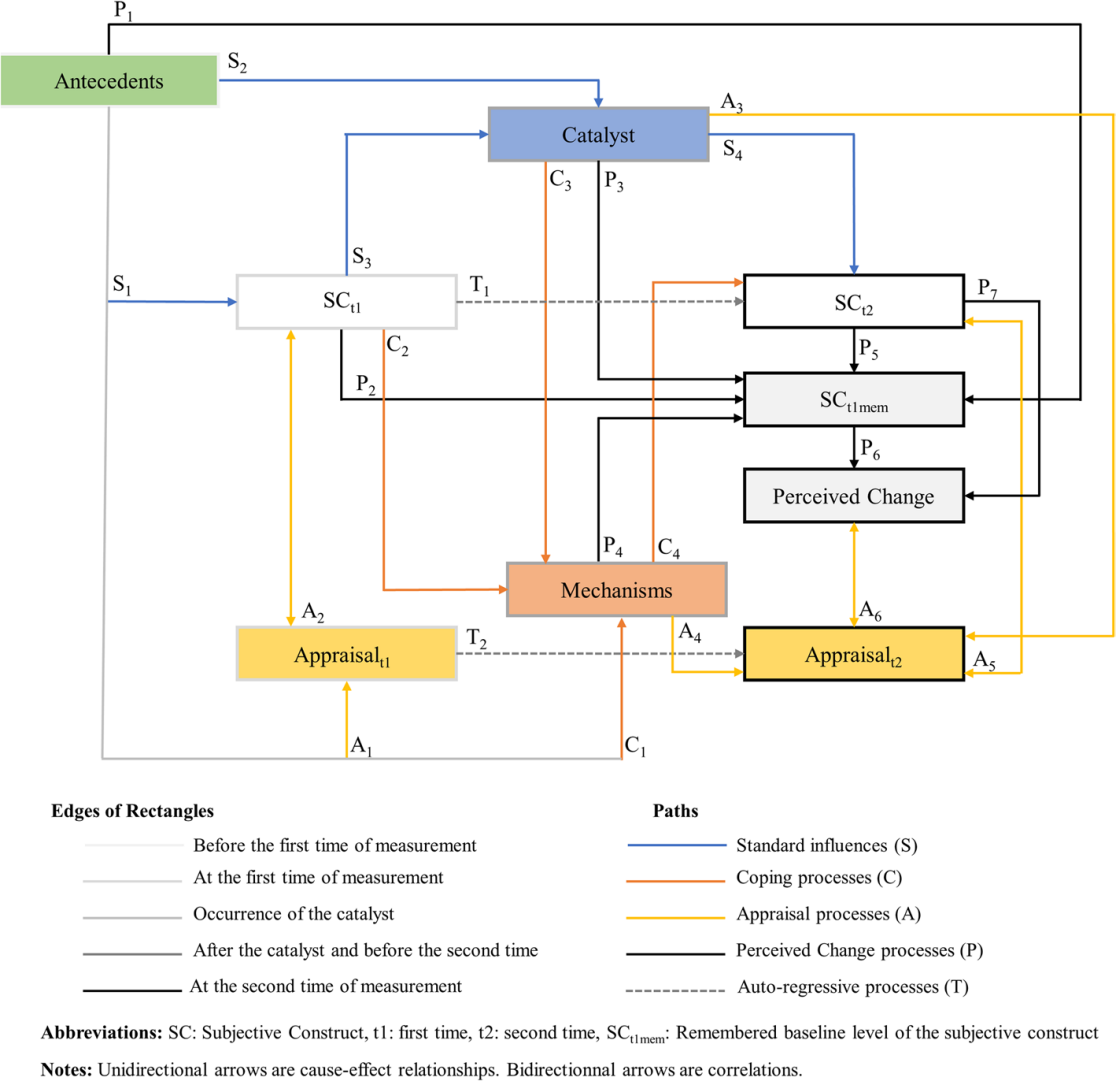
A theoretical model depicting the components engaged when someone must rate his/her level on a given PRO at two measurement times and must answer a PGRC at the second time

The final conceptual model is depicted in Fig. 3. A breakdown of all the included and nonincluded paths is depicted in Supplementary Appendix 2. Based on the proposed conceptual model, we hypothesize that perceived change is the cognitive concept measured by a PGRC at the second measurement *(A*_*6*_ path).

### A second proposal: parameterizing the minimally perceived change in the population

We have proposed that a PGRC is an item that measures a construct we called Perceived Change. It is therefore necessary to explicitly define the MPC as a statistical parameter. To achieve this, we present several assumptions.

**Fig. 4:**
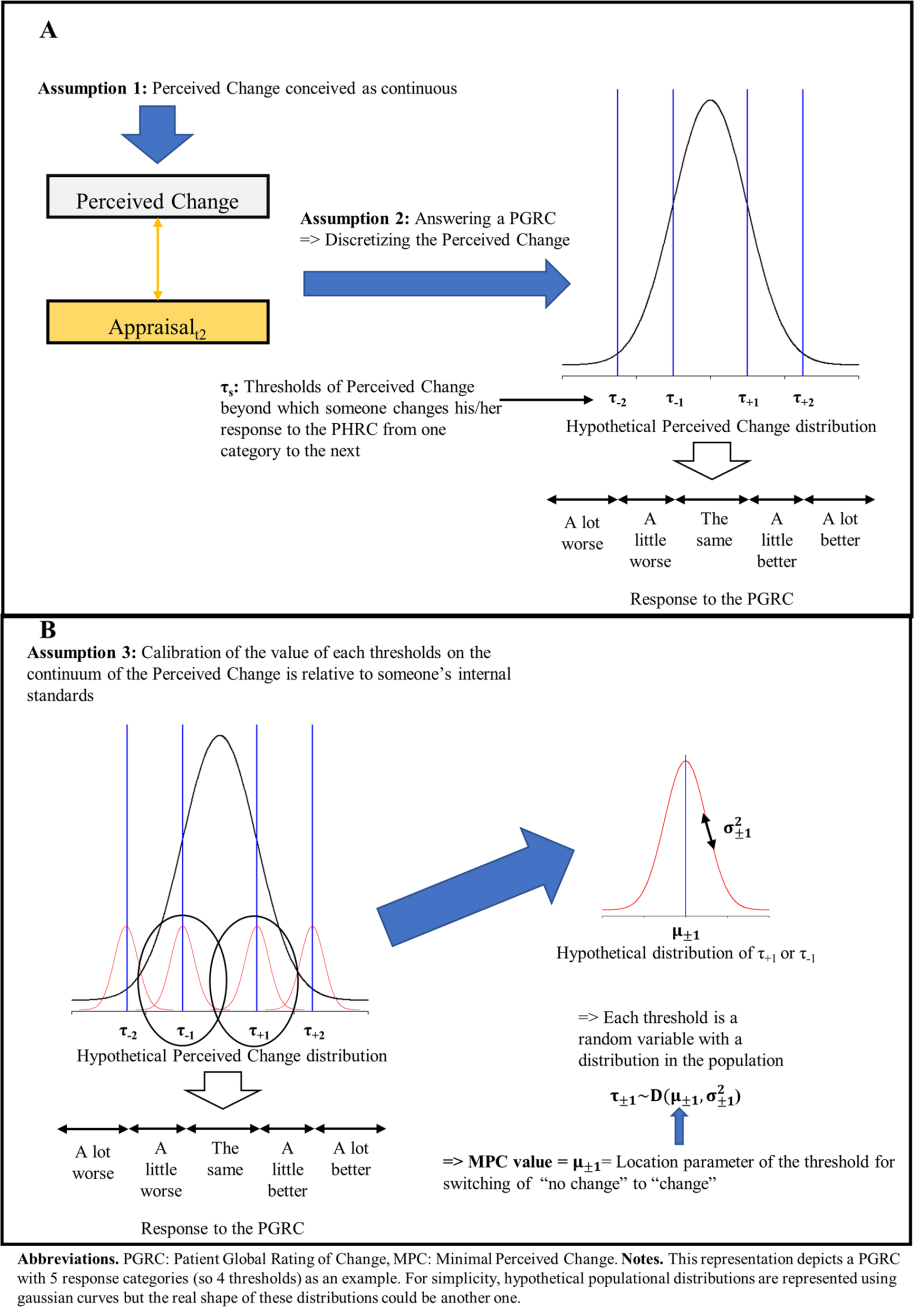
Toward a definition of the minimal perceived change as a statistical parameter in the population. **a**: The measurement of perceived change by a patient global rating of change. **b**: Defining the value of the minimal perceived change

First, we assume that when someone is asked to rate his/her PC, this hypothesized construct corresponds to a variable that can be primarily conceived as mapped onto a continuous scale. Regardless of the “true” link between the cognitive processes engaged when one must rate his/her level on a target construct and the mathematical objects used to operationalize them, such as variables, we adopt a pragmatic approach that is in agreement with the usual operationalization in most measurement models used in psychometrics [8]. In *Classical Test Theory*, constructs of interest are most frequently measured by a variable called the *score*, which is conceived as continuous [18]. In models with latent variables (i.e., *Rasch Measurement Theory*, *Item Response Theory*, *Structural Equation Modeling*), the variable measuring the construct is on the real line [2]. In our model, the PC is the difference between two constructs operationalized as continuous. An advantage of considering the PC as a construct that can be mapped onto a continuous scale is that we can parameterize its distribution using a continuous density function such as the normal distribution (see Fig. 4, where its distribution is represented as such for illustration) with two parameters (i.e., location and dispersion parameter) that synthesize most of the statistical information.

Second, we assume that answering a PGRC (which usually proposes 5 to 7 response categories) is equivalent to asking the patient to discretize a PC conceived as continuous into one discrete state among the proposed response categories. Therefore, we propose two cognitive processes that are engaged when people appraise their level of PC through the response to a PGRC. First, the patient needs to set several thresholds of PC values on the PC scale (we denote them τ_s_ (for a PGRC with *k* response categories, there are *k*-1 τ_s_); see Fig. 4a). These thresholds are critical values that define the bounds for switching from one response category (e.g., “the same”) to the next (e.g., “a little better”). Second, the patient needs to compare the level of PC with adjacent τ_s_ to provide the appropriate response on the discrete PGRC scale.

Third, we assume the calibration of the value of both the continuous PC scale and each threshold τ_s_ on the continuum of the PC is relative to the individual’s internal standards. For example, each patient can have his/her own internal calibration of the threshold values discriminating the passage from the “about the same” response category of the PGRC to the “a little better” response category. Thus, for a given threshold, we cannot expect the value to be identical among the patients of a population of interest. For a PGRC with *k* response categories, each *k*-1 threshold τ_s_ can be considered a random variable with a distribution in the population (Fig. 4b). This implies that for the same level of PC, an individual can answer differently from another for the PGRC.

If we denote τ_1_ as the threshold of the PC value representing the shift from “about the same” to “a little better” and τ_-1_ as the threshold of the PC value representing the shift from “about the same” to “a little worse”, then these two thresholds are random continuous variables with a distribution that can be parameterized with a location and a dispersion parameter:

$${\uptau }_{1}\sim \mathrm{D}\left({\upmu }_{1},{\upsigma }_{1}^{2}\right)$$

and

$${\uptau }_{-1}\sim \mathrm{D}\left({\upmu }_{-1},{\upsigma }_{-1}^{2}\right).$$

It follows that the MPC for improvement (MPC_+_) and the MPC for deterioration (MPC_-_) are the values of the location parameter of the distribution in the populations τ_1_ and τ_-1_, respectively:

$${\mathrm{MPC}}_{+}={\upmu }_{1}$$

and

$${\mathrm{MPC}}_{-}={\upmu }_{-1}.$$

We note that we make this distinction between the MPC for improvement and the MPC for deterioration because the current view on obtaining an MID value assumes that the values for improvement and deterioration are not symmetrical relative to 0 [30].

### A third proposal: hypotheses about appropriate estimator(s) of the MPC

According to our model, the MPC is the location parameter in the population of a threshold value discriminating the PC from a shift to a range of values considered no change to a range of values considered either improvement or deterioration. Therefore, estimating the MPC is about finding a threshold that best discriminates people who perceive themselves as having not changed from those who perceive themselves as either improved (for the MPC_+_) or deteriorated (for the MPC_-_).

Multiple estimators using anchor-based methods with the PGRC as the anchor question have been proposed in the literature. A fundamental note is that the link in this setting anchors the change in the PRO scores to the answer to the PGRC; these are the data used in practice for the estimation of the MCID or MID [24]. However, in empirical data, there is never a quantitative measure on a continuous scale of the PC. This implies that an estimation of the MPC value will be an adequate representation of the threshold value to be searched only if there is a sufficient level of correlation between the change in the PRO scores and the PC. The ideal case would be a perfect correlation. As illustrated by Fig. 3, for instance, this ideal scenario could occur if the level of SC_t1mem_ is a perfectly accurate match to the level of SC_t1_ (e.g., perfect recovery of the level at baseline of the construct of interest (P_2_ path), with the other contingencies depicted by the P_1_, P_3_, and P_4_ paths not altering this perfect recovery). Even without assuming this ideal case, this issue has been illustrated in the literature about anchor-based methods, and several authors have noted that an MID should only be estimated if a correlation that is at least moderate between the answer to the PGRC and the change in PRO scores has been demonstrated in the data [24].

**Fig. 5:**
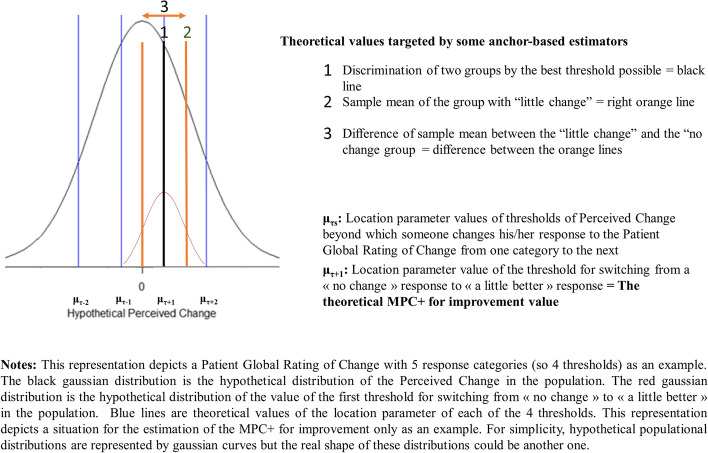
Theoretical values of perceived change targeted by anchor-based estimators

Two of the most commonly used anchor-based estimators rely on the distribution of the change in PRO scores in the subgroups of patients who answer that they have experienced little change (either improvement or deterioration). The first estimates the sample mean of the change in the PRO score as a plausible MID value. The second is the difference between the sample mean of the change in the PRO scores in the group with little change and no change. Assuming a sufficient correlation between the change in the PRO scores and the PC and according to our model, these estimates do not seem to be the most appropriate since they target values in the neighborhood of the theoretical MPC but not the MPC threshold itself (Fig. 5).

Another type of frequently used anchor-based estimator relies on finding a threshold that best classifies people with little change or no change with a minimum amount of classification errors. A popular way of doing this is to plot a *Receiver Operating Characteristic curve* (ROC curve). The threshold value can be selected with a classification criterion such as minimizing the Euclidian distance with the top left point of the ROC Cartesian diagram [24]. However, depending on the studied context, criteria that give more weight to sensitivity or specificity can be applied [36]. Other methods relying on the discrimination of different subpopulations can also be used [29, 36]. We believe that these discrimination techniques attempt to successfully target the theoretical MPC threshold.

In general, according to our model, the issue of estimating the MPC is an issue of discriminating the best three subpopulations by finding two appropriate threshold values (MPC_+_ and MPC_-_). Thus, we can assume that the essential data to collect are information about whether people in the sample have experienced a trajectory over time on the target construct they considered a change (either improvement or deterioration). We hypothesize that the simplest way to obtain this sufficient level of information is to use a PGRC with only three response categories. It could be phrased as follows: “Since [adequate reference point], do you think your overall [construct of interest] is…”, with the proposed responses of “worse”, “the same”, and “better”. A PGRC with more response categories can be useful to discriminate more subpopulations (such as obtaining a threshold of change in PRO scores discriminating “little” change from “large” change). However, from the perspective of obtaining an MPC, this simple format should be sufficient and ensures no additional complexity in the interpretation of calibration adjectives such as “a little” or “a lot” [35].

As mentioned above, we assume that answering a PGRC implies evaluating the value of thresholds that discretize a continuous PC in a person’s mind. According to our model, the thresholds of PC values are formed because a patient is asked to answer a PGRC. That is, the elicitation of the cognitive processes leading to the existence of these thresholds is a direct consequence of asking the patient to do so by means of answering the PGRC. Moreover, the values of the thresholds chosen by a patient are relative to his/her subjective experience and internal standard. In this context, since distribution-based methods such as the use of Effect Size do not elicit those cognitive processes, it makes no sense to assume that an estimate of the MPC using such estimators targets the theoretical MPC. In the absence of explicit hypotheses about a dependency between a distribution-based estimate and the theoretical MPC, if a distribution-based estimate matches the theoretical MPC value, it would likely be by chance only. Therefore, this deduction from our model is in line with authors who state that distribution-based methods cannot derive an MID value according to the patient’s perspective but rather are complementary methods for the interpretability of changes in PROs according to other perspectives [30].

### How the model and definition can be used to plan experimental studies on the statistical properties of estimators of the MPC

Assuming appropriate statistical modeling, our conceptual model and formal definition of the MPC can now be theoretical bases for sound *Monte Carlo simulation studies* investigating the statistical properties of numerous methods used to estimate an MPC. Each component can be operationalized by random variable(s) (latent or manifest) with hypothesized distributions, and the proposed pathways between the components can be operationalized as mathematical functions to derive appropriate values.

For a sample of a given size, responses to items of a hypothetical questionnaire can be simulated under the constraint of known distributions of SC_t1_ and SC_t2_ using an appropriate psychometric model. Then, for each individual, the value of SC_t1mem_ can be derived as a function of SC_t1_, SC_t2_, and other contingencies (catalyst, antecedents, etc.). Different combinations of coefficients weighting the importance of SC_t1_, SC_t2_ and other contingencies in explaining the value of SC_t1mem_ can be used to test different scenarios (such as the value of SC_t1mem_ as a perfect recovery of the value of SC_t1_ or a value of SC_t1mem_ heavily reconstructed from the value of SC_t2_). From there, the value of the perceived change can be derived as the difference between SC_t2_ and SC_t1mem_. The different combinations of the aforementioned coefficients explain the correlation between the change in the construct (SC_t2_—SC_t1_) and perceived change. Finally, for each individual, assuming a known distribution of ${\uptau }_{1}$ and ${\uptau }_{-1}$, the PC can be discretized into a response to a PGRC under the constraint of a known populational MPC value for improvement and deterioration. After simulation of the data (responses to the questionnaire at two measurement times and response to a PGRC), these data can be used to estimate MPC values using various methods, and the bias of the different methods can be estimated under different scenarios (sample size, characteristics of the PRO questionnaire, influence of recall bias, characteristics of the PGRC, specific characteristics of discrimination techniques such as the rule to define a threshold value, etc.).

## Discussion

In this paper, we propose for the first time a comprehensive model leading to a formal definition as a statistical parameter of an MID, which we call the MPC, in the context of the interpretation of PRO scores over time in health-related research and practice. Here, the MPC is formally defined as the location parameter value of the populational distribution of the threshold discretizing the Perceived Change from a perception of no change to change (either improvement or deterioration). This discretization is elicited by answering a PGRC item. We believe that this theoretical proposal is potentially a valuable addition to the literature about the interpretation of changes in PRO scores because it formally defines a parameter that has been estimated hundreds of times in empirical data from clinical samples [36]. Thus, we believe that this definition can satisfy a fundamental prerequisite in estimation theory, which is the theoretical definition of a parameter in the population, and it can help to alleviate the lack of clarity in this research field.

Nonetheless, at this stage, this model is an initial theoretical proposition only. As such, obvious potential limits are the assumptions made to develop the definition of the MPC. The purpose of this model is to serve as a useful theoretical framework to foster future advances in interpreting changes in PRO scores, which is an issue often encountered in clinical research and healthcare practice. Thus, our approach here is mostly pragmatic realist: our model attempts to bridge concepts from cognitive psychology, psychometrics and statistical modeling in clinical research. Thus, we have adopted representations in line with those usually relevant in these fields, such as the idea of defining hypothesized cognitive constructs (e.g., the PC, the SC_t1mem_) that can be measured quantitatively by operationalization as variables on a continuous scale [8]. However, we need to explicitly state that the soundness of these theoretical objects as adequately representing the cognitive processes at play in this context is a conjecture.

On a more specific note about conjecture in modeling, our model is a Structural Equation Metamodel and makes no assumptions about the mathematical functions linking the different components. Nonetheless, to progress, we described the variables used in the model as scaled on a continuum. These constructs can be operationalized as random variables. For simplicity (see Figs. 4 and 5), we assumed normal distributions, as are usual regarding the shape of the distribution of many hypothesized constructs relevant in psychometrics and health sciences [2]. Nonetheless, we again need to state that this assumption is a conjecture, and the most adequate shape to model the distribution of constructs we have introduced in the model could be scrutinized in more depth.

We based our model on the Rapkin and Schwartz appraisal model [22]. This appraisal model was also grounded on the seminal work of Tourangeau et al. about the psychology of survey responses [31]. Since the inception of the appraisal model in 2004, different instruments to measure the appraisal processes at play when a respondent is answering a PRO have been developed and used to investigate the role of the change in the meaning of a target construct over time in the interpretation of change in PRO scores [20, 21]. The use of this appraisal model on empirical data sheds new light on the determinants of changes in PRO scores and psychological adaptation to illness [11, 27, 28]. Nonetheless, to date, all components of the model have not been modeled together on empirical data by means of statistical techniques such as Structural Equation Modeling. This is probably related to the fact that a full investigation of the model would require a complex collection of highly standardized clinical data on a large sample of people with many PROs. Thus, a limitation of our work is that we based our model on a theory whose fit must be investigated further with empirical data to be fully validated. However, we also note that Figure 2A provides a direct representation of the theoretical model that could be tested on data with measures at two times.

Our model relies on a PGRC as the anchor question. In recent years, the use of two cross-sectional Patient Global Ratings of Severity (PGRS, i.e., an overall impression of the severity of the disease at each time of measurement) has been advocated as more appropriate anchors because these are not susceptible to recall bias compared to a PGRC [33]. While our model acknowledges that a PGRC is susceptible to recall bias, we argue that this type of anchor is nevertheless an assessment of perceived change according to the patient’s perspective. To estimate an MID value using two cross-sectional PGRS, the difference between the two is estimated, and then a rule is applied by a health professional to define the minimum change (such as a difference of one point between the two assessments). Thus, when using two PGRS, 1) the difference between the two is an overall measure of actual change in the construct of interest, not perceived change, and 2) the definition of a minimum change is relative to a choice made by the health professional, not the patient (i.e., the perspective is not the patient’s perspective) [34].

A last limitation is the scope of our model. We attempted to develop a definition of RD according to the patient’s perspective of what he/she subjectively considers a change. This is just one perspective and does not include all useful perspectives (e.g., the societal perspective) that can be invoked when attempting to interpret the relevance of a change in PRO scores [1].

## Conclusion

Our work, with its formal and explicit definition of the MPC and a model describing how this MPC value can be determined, can serve as a basis for experimental studies of the statistical properties of the numerous estimators that have been proposed to estimate the MPC against an explicitly defined population value. Within our research group, we have derived a simulation model with specifications fitting the conceptual model proposed in this paper and have designed a large simulation study to investigate the statistical performance (especially bias) of numerous MID estimators proposed in the literature under various scenarios. Analyses of the results of the simulation are underway.

The development of new methods can be facilitated because there is now a model describing the psychological processes leading to the elicitation of this MPC value in people’s minds. We propose in our model that thresholds of PC values are random variables. The MPC is defined here as the location parameter only of τ_1_ or τ_-1_. This means that if we restrict an estimate of the MPC as a location parameter only, we may lose relevant data about the dispersion of the thresholds of minimum PC values corresponding to changes in the population of interest. Therefore, a new method to estimate an MPC value as a random variable with location and dispersion parameters could be a relevant development.

## Data Availability

Not Applicable.

## Abbreviations

ES: Effect Size
FDA: US Food and Drug Administration
HRQoL: Health-Related Quality of Life
MCID: Minimal Clinically Important Difference
MID: Minimal Important Difference
MPC: Minimal Perceived Change
PC: Perceived Change
PGRC: Patient Global Rating of Change
PRO: Patient-Reported Outcomes
PRO: Patient-Reported Outcomes Measure
RD: Responder Definition
ROC: Receiver Operating Characteristic Curve
SEM: Standard Error of Measurement
